# Artificial Intelligence, Medical Knowledge, and Empowering Patients

**DOI:** 10.1016/j.mcpdig.2024.01.008

**Published:** 2024-02-28

**Authors:** Allen O. Eghrari

**Affiliations:** Johns Hopkins University School of Medicine, Baltimore, MD

It is the night after your emergency eye operation and the alarm on your mobile phone suddenly jolts you awake. Tired but diligent about getting each drop in, you carefully remove the protective shield to administer the next dose. As you catch a glimpse of your eye in the mirror, you realize it is much more swollen than you anticipated. Fearful of an infection, you find the emergency number and rapidly begin dialing, your fingers struggling to reach the right keys.

Hoping to share your predicament with the on-call physician, the operator tells you that the physician is currently on the line with another patient, but an automated chat service is listening and can answer your questions instead. Restless for answers, you agree and begin to state your concerns. The chat service summarizes them in an organized manner and initiates a reply. At that moment, you are grateful for any response, but what qualities would characterize an appropriate response?

As clinical practices across the world seek to harness the power of generative artificial intelligence (AI) to provide automated, empathetic responses to patients’ queries,^1^ these questions and scenarios require our consideration.

Generative AI refers to algorithms that learn from data using machine learning techniques and output new content, such as text or images. A relevant step forward in the widespread adoption of such technologies occurred in November 2022, when the public release of chat generative pre-trained transformer (ChatGPT) (OpenAI) brought significant attention to the capabilities of large language models (LLMs). These algorithms are trained on vast quantities of internet text, including books and websites. ChatGPT is the most well-known of these, reaching 100 million regular weekly users by November 2023.^2^ As an LLM, ChatGPT does not fully understand a concept in a general sense, but generates text by calculating and choosing the most probable next word based on the context provided by the preceding words. ChatGPT’s abilities to chat with users, provide information, and generate creative works from poetry to code have improved across updates and iterations, as has its fund of ophthalmic knowledge.^3^

It is not surprising, then, that clinical practices faced with both financial and staffing constraints are turning to LLMs, such as ChatGPT, to provide patients with knowledge about diseases and information about operation,^4^ and patients increasingly seek out information on such topics. Although health-related LLMs, such as Med-PaLM (Google) can answer medical questions effectively,^5^ ChatGPT is still the model most widely used by the public. By answering patient questions, these models can potentially transform engagement with medical knowledge from a one-way presentation to a two-way conversation.

However, all technologies require validation, and the extent to which ChatGPT can describe nuances around eye conditions and their treatment needs to be rigorously tested. Although ophthalmic diseases and therapies are constantly evolving, data in the model are supplemented periodically and may not have been recently updated; at the time of this writing, the latest training cycle was 9 months ago. Given the above, how capable is ChatGPT of providing fully appropriate information to patients?

In “Appropriateness of ophthalmology recommendations from an online chat-based AI model”, Tailor et al^6^ address this point, performing a cross-sectional study that included 22 subspecialists across a variety of fields of ophthalmology, with each grader entering approximately 20 subspecialty-specific questions into ChatGPT 3 times. They assessed whether its responses were appropriate based on 2 possible contexts: information on a patient-focused institutional website, and information from a response to patients in the electronic medical record.

A strength of this study was its collaborative approach with many investigators from multiple subspecialties assessing responses in parallel: cataract or refractive, cornea, glaucoma, neuro-ophthalmology, vitreoretinal diseases, uveitis, oculoplastic and orbital operation, pediatric ophthalmology, and ocular oncology.

The authors found that 79% and 74% of answers in these 2 contexts, respectively, were deemed appropriate, with a wide range across subspecialties. For example, in contrast to the 100% of ocular oncology responses that were favorably reviewed in the first context, only 56% of cornea responses were considered appropriate. Although these differences did not achieve statistical significance, preventing us from specifying which subspecialties may be more susceptible to accuracy or error, the broad range indicates the need for further investigation into this area.

What is necessary for a response to be considered appropriate? Indeed, to empower patients with knowledge, effective responses must offer more than simply the presentation of correct facts. For example, validation studies of Med-PaLM assessed consistency with the scientific consensus, the presence and clinical significance of omissions, the presence of inappropriate or incorrect content, the extent and likelihood of possible harm, and the possibility of bias,^5^ all of which could still be associated with the presence of correct facts. An advantage of assessing a broad term, such as appropriateness as an outcome in this study is that responses must essentially succeed at all of these tasks to be considered appropriate. The methodology used in this study would therefore be expected to have high specificity for appropriate answers.

Although it is encouraging, therefore, that this algorithm is capable of providing mostly appropriate answers, analysis of the inappropriate answers is equally important. In this study, the authors identified questions related to operation as a particular weak point, resulting in a high frequency of inappropriate responses compared with general information about diseases and conditions. These results are consistent with data from a similar study in which general information provided by ChatGPT about a corneal dystrophy was rated highly, but specific information about its surgical treatment was inaccurate and even dangerous if followed.^7^

An implication of the above findings is that responses from ChatGPT or other LLMs still require moderation or supervision by clinicians. Such models are susceptible to hallucinations, made-up facts that can be at times expressed with confidence because the words provided are based on probability and context. For example, in the aforementioned study, ChatGPT erroneously responded to a query about cornea transplants by suggesting that the questioner could become a living donor, creating a convincing but nonexisting citation to justify its response.^7^ However, studies have shown improvement in quality of information across generations of LLMs,^3^^,^^7^ and given the ability of models to learn, the number of necessary corrections will likely decrease over time across updates. However, if some responses carry risk of harm, then the ethical principle of nonmaleficence becomes particularly relevant.

In addition, relevant is the ethical principle of justice, and it is hoped that as LLMs improve in quality, they will allow increased access to knowledge in areas without direct access to physicians. However, if the data set or graders providing feedback are isolated to a specific population, the responses can be inherently or gradually more biased over time. The diversity of gender and ethnicity among the team of collaborators adds strength to the study by Tailor et al^6^ and should be considered, along with patient perspectives, in methods of validating new technologies as they arise.

Applications of AI in ophthalmology are numerous, and apart from generative AI, which creates content, important advances have been made in diagnostic AI, in which machine learning is used to identify patterns, anomalies, or signs of disease from inputs such as retinal scans.^8^ However, applications of these technologies do not need to be mutually exclusive, and a future area of learning and research will be the intersection between generative and diagnostic AI. For example, can AI-based conversations engage with a patient’s requests for information on a website, or reply to messages to a provider in the electronic health record, the 2 contexts studied here, to determine a diagnosis? Could an AI algorithm triage a patient based on an uploaded medical image, and ask appropriate follow-up questions? Although data are limited in this area, early studies are promising.^9^^,^^10^

The near future of AI will include more choices of LLMs, more users, and more knowledge for training algorithms. Research in this area will help optimize such models to empower patients more effectively.

## Potential Competing Interests

The authors declare no conflict of interest.
